# Supplementary material for: Analysis of fatality impact and seroprevalence surveys in a community sustaining a SARS-CoV-2 superspreading event
Source: Sci Rep. 2023 Apr 3;13:5440. doi: 10.1038/s41598-023-32441-7 (PMC10069345; doi:10.1038/s41598-023-32441-7)
Supplement: Supplementary file 1 — Supplementary Information. [file 41598_2023_32441_MOESM1_ESM.docx]

**Supplementary Methods**

*Statistical analysis*

To account for possible uncertainty in the number of SARS-CoV-2-associated deaths we use a Bayesian credibility interval for the IFR which, however, can only be interpreted for populations for which the full survey in the community means a representative sample. This CI was computed as the empirical 2.5% and 97.5% quantiles of 100,000 samples drawn from a a-posteriori beta distribution with parameters α = [SARS-CoV2-associated deaths] + 1 and β = [estimated number of infected] - 7 + 1. This a-posteriori distribution results from a binomial likelihood model for the number of SARS-CoV-2-associated deaths with parameters trials = [estimated number of infected] and successes = [SARS-CoV-2-associated deaths], and an uninformative uniform prior distribution for the IFR. To account for uncertainty in the number of infected, we use Monte-Carlo integration by sampling the estimated number of infected, for each of the 100,000 samples, form a Gaussian distribution with mean 0.153 and standard deviation 0.016 multiplied by 12,597 (i.e. the number in inhabitants in the community, January 1, 2020).

**Supplementary Materials**

**Supplementary Table 1** Deaths per month and age stratum from the 77 deaths we were able to examine


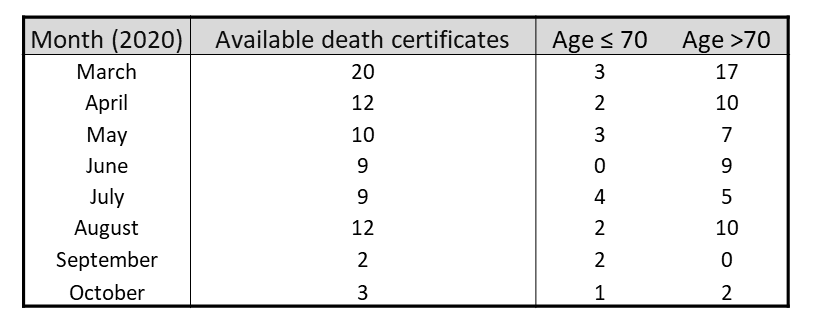


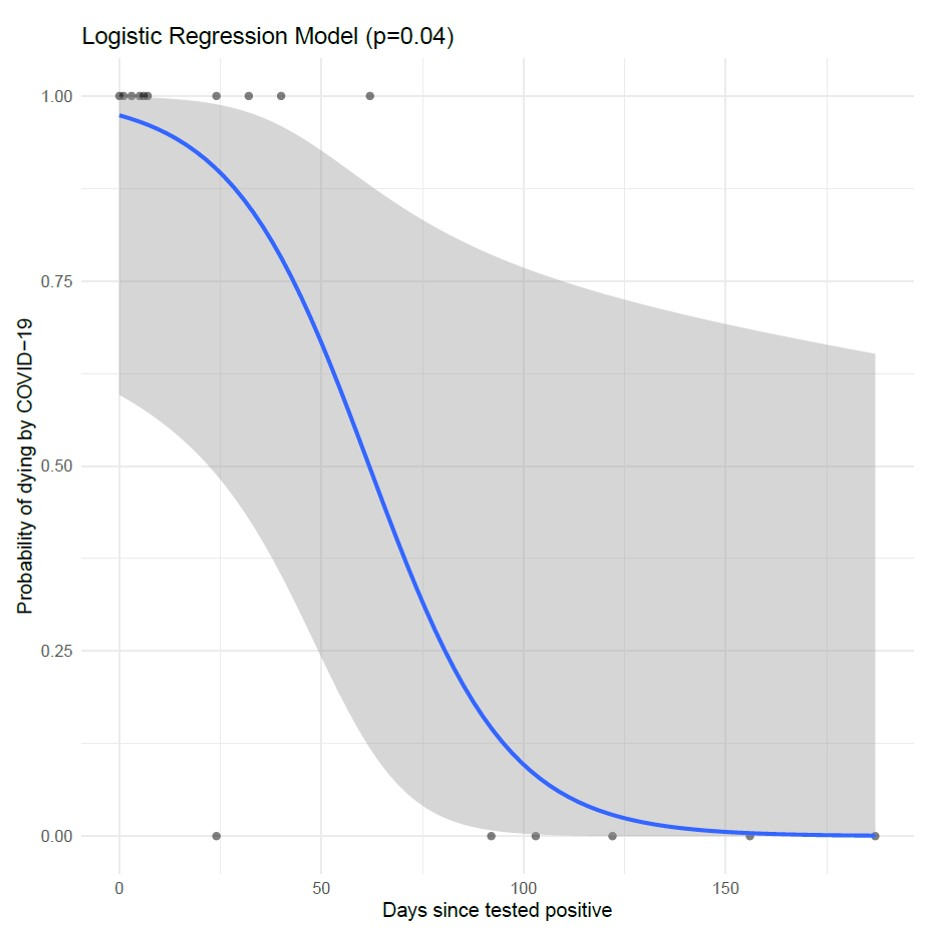


**Supplementary Fig. 1** The likelihood to die at COVID-19 underlying causes of death after positive PCR-test for severely ill individuals in the specific community was examined. A logistic progression model demonstrating that the likelihood to die of COVID-19 is significantly decreased (p=0.04), if death occurred 100 days or later after confirmation of SARS-CoV-2 infection.
